# Failure gas analysis of lithium–nickel–cobalt–aluminium oxide cells from different manufacturers

**DOI:** 10.1039/d4ra07884e

**Published:** 2025-02-18

**Authors:** Philip A. P. Reeve, Jonathan E. H. Buston, Jason Gill, Steven L. Goddard, Gemma E. Howard, Jack W. Mellor

**Affiliations:** a HSE Science and Research Centre Harpur Hill Buxton Derbyshire SK17 9JN UK philip.reeve@hse.gov.uk jonathan.buston@hse.gov.uk

## Abstract

Lithium ion batteries (LIBs) are now commonplace industrially and domestically, innovations in their size and capability in terms of charge and discharge rates also mean LIB applications are growing. LIBs also present a unique challenge when the undesirable happens and they fail. One of the motifs of catastrophic LIB failure is the production of large volumes of flammable and toxic gas. Characterising LIB failure and the products of such events is an area of significant interest. In this work an array of nickel–cobalt–aluminium oxide (NCA) LIBs from four different manufacturers were failed predominantly by external heating but also by nail penetration. 18 permutations based on cell type and amounts of charge (69 tests in total) have been reported. Failure was carried out in inert atmospheres of nitrogen or argon inside a sealed vessel. After LIB failure, gas samples were taken, the volume calculated and the relative amounts of CO_2_, CO, H_2_, CH_4_, C_2_H_6_, C_2_H_4_, C_3_H_8_ and C_3_H_6_ determined using mass spectrometry. The volume of gas produced during LIB failure by each cell type at 100% state of charge (SoC) has been analysed and is reported in the range of 1.34–2.32 L Ah^−1^ for cells between 2 and 5 Ah sourced across four manufacturers. The volume of gas produced by LIB failure at differing amount of charge (AoC) has been determined for 2, 3, 4 and 5 Ah cells sourced from a single manufacturer. Variations in the volume of gas produced are shown to not only be dependent on AoC but also the type of cell has a material effect on this aspect of LIB failure. This work supports the existing consensus that as AoC increases, so does the volume of gas released as a result of LIB failure. In terms of gas composition a general trend of increase in flammable components and decrease in CO_2_ once SoC is >50% has been observed in this dataset. This work also demonstrates that whilst LIB failure can produce some interesting phenomena, understanding and ultimately predicting the outcomes of LIB failure is difficult. The variations reported, even within a single cell manufacturer, suggests that for safety critical applications relying on generic or typical values is less useful than testing the precise cell being considered.

## Abstract 2

Cells with the same cathode chemistry do not necessarily fail in the same way.

## Graphical abstract

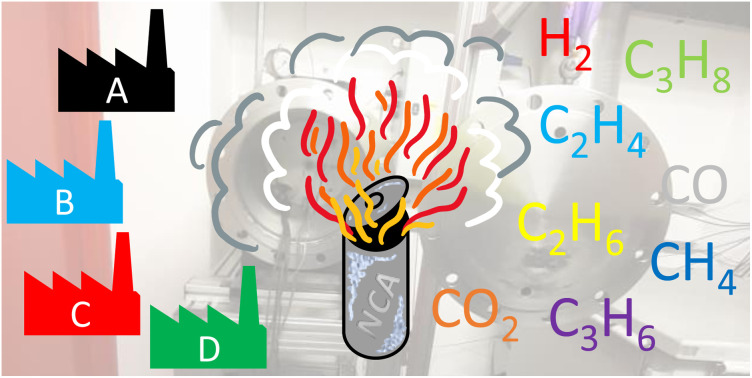

## Introduction

### Lithium ion batteries (LIBs)

Lithium ion batteries (LIBs) are now ubiquitous and form essential components in portable electronics, electric vehicles (EVs), as well as domestic, commercial and grid based energy storage solutions. As the world acts to meet Net Zero targets LIBs are set to perform a vital role in energy storage and delivery.^1^ The capacity and charge/discharge properties of LIBs can vary depending on their desired application. One of the main determinants for cell characteristics is cathode chemistry. However LIBs also include anodes, binders, solvents and other additives that allow the cell to function as intended. Because much of the construction of LIBs is commercially sensitive, the precise make-up of the interior of LIBs cannot be deduced without advanced break down and analytical techniques. Usually, all that is known are the physical dimensions and operational characteristics in terms of capacity, voltage, optimal and maximum charge/discharge parameters.

### LIB failure

When subjected to aberrant conditions, LIBs can fail. LIB failure events are often violent and can produce fumes, flames and fast moving debris. These events pose significant risks to workers, members of the public and first responders.^2,3^ Due to the present ubiquity of LIBs in small electronics, electric vehicles (EVs) and battery energy storage solutions (BESS) and the forecast increase in the number, size and capacity of LIBs in all areas, it is important in assuring safety that phenomena produced by LIB failures are understood to the fullest.^3–7^

LIB failure is usually caused by three general types of abuse; mechanical, electrical and thermal. The failure of LIBs can be as a result of accidental or intentional abuse. Defects resulting from improper manufacture can also be a source of LIB failure. When pushed physically, thermally or electrically LIBs can enter thermal runaway (TR). The first stages of LIB failure may be gradual, however there does reach a point when the degradation processes inside LIBs become rapidly self-heating. This results in fast increases in temperature and LIBs may vent combustible and toxic gases and smoke,^8,9^ vent gases can ignite,^10,11^ LIB casings can glow red hot, contents may be ejected^12^ and cell debris can become a projectile hazard.^13^ In incidents involving LIB failure, the degradation processes that lead to TR often occur unseen and unnoticed. In the case of modules that contain multiple cells, one undergoing TR may cause others to also fail, compounding the hazards present.^14^

**Fig. 1:**
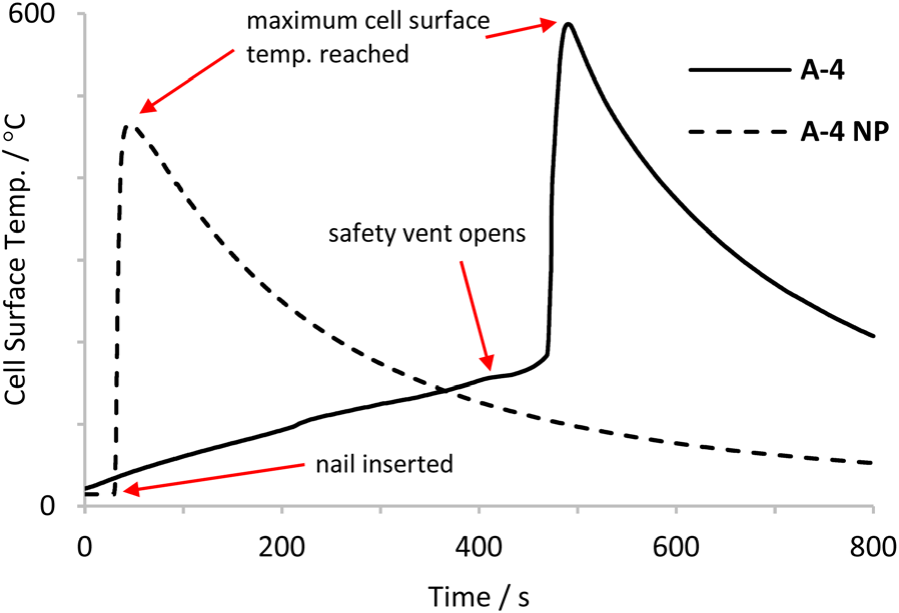
Plot of cell surface temp. against time as LIB A-4 is heated to failure and penetrated with a reinforced stainless steel nail (A-4 NP).

In this work cells have been deliberately made to undergo TR. The majority of cell failures here were achieved by external heating. An example of the usual profile of cell failure by external heat is shown with respect to cell surface temperature (Fig. 1). Heating causes internal components of LIBs to degrade, this releases gases and at a certain internal pressure the safety vent opens (venting). The safety vent opening usually results in a slight cooling of the cell surface as vapour and gases expand. There is then typically a time delay between venting and the onset of rapid cell failure. However cells do not always follow this heating profile and may enter TR without any prior visual cues. Determining the exact point TR begins is difficult as there are many processes occurring simultaneously at different rates which contribute to TR.

LIB failure and the associated hazards have been described in the literature. In previous work at the Health and Safety Executive (HSE) three nickel based cells were tested and their behaviour during cell failure was probed. In 2022 Abbott *et al.* investigated the timing of the events outlined in Fig. 1 and how LIB failure by heating was affected by state of charge (SoC). Also in that work the vent gases produced by LIB failure were investigated and characterised by real time gas analysis, total gas volume and overall composition when LIBs failed in both air and in nitrogen atmospheres.^13^

### Nickel cobalt aluminium oxide (NCA) cells

There are several cathode chemistries available to LIB manufacturers. Common varieties of cells include lithium iron phosphate (LFP), nickel manganese cobalt oxide (NMC) and nickel cobalt aluminium oxide (NCA). Varying cathode chemistry is a tool used to modify the performance characteristics of LIBs. Probing the differences in behaviours during abuse of LIBs with differing cathode chemistries has been the subject of several studies. Golubkov *et al.* (2014),^15^ Sturk *et al.* (2019)^16^ and Mao *et al.* (2023)^17^ are examples of work comparing failure characteristics of nickel based LIBs and LFP LIBs. LFP cells tend to be considered as less aggressive in their failure due to the higher energy density of nickel based cells.^18^ However, less aggressive failure comes at a notable cost to performance with LFP cells having lower energy densities compared to nickel based alternatives.

In this work all the cells studied have NCA cathode chemistry in order to probe variations in LIB failure between cells of the same cathode chemistry. The ratios of nickel : cobalt : aluminium in NCA cell cathodes are not disclosed by manufacturers, if cathode composition is disclosed at all. A typical composition is LiNi_0.8_Co_0.15_Al_0.05_O_2_. However, this varies and results in different energy densities for NCA based LIBs.^19^ In 2018 Ping *et al.* published work characterising the release of this stored energy using calorimetry techniques.^10^ Most of the work relating to the abuse of NCA cells has most commonly used heating as the method of abuse.^20^ Lalinde *et al.* recently (2023) investigated the interplay of heating and overcharge in combination when contributing to LIB failure.^21^ Whilst there have been a number of reports concerning the characteristics of NMC cell failure, there are comparatively fewer for NCA cells.

In 2015 Golubkov *et al.* studied the vent gases from TR of a single NCA cell, alongside an LFP cell.^22^ The cells were abused by heating at varying SoC (0–143%) and the gases evolved analysed by GC. For NCA cells, it was observed that the composition of gases changed with increasing SoC. At 0% SoC there was found to be negligible H_2_, CO, CH_4_, C_2_H_4_ and C_2_H_6_, the bulk of the gas (>94%) was CO_2_. The next highest SoC was 25%, at this point the compositions of H_2_ jumped to 15.5%, CO_2_ decreased to 62.7%, CO increased to 5.5% from 1 to 2%. CH_4_ increased to 8.7% from 1.1 to 1.6%, C_2_H_4_ increased to 7.5% from 0.2 to 0.4% and C_2_H_6_ remained negligible but was observed at higher SoC. At 100% SoC H_2_ gradually increased to a maximum of 28.5%, CO_2_ decreased to a minimum value of 17.5%. The hydrocarbon content remained comparable to that of 25%. The analysis of gases at >100% SoC was comparable to that of 100% SoC. The volume of gas produced also increased up to 100%, then appeared to plateau, but produced fluctuating volumes at ≥100% SoC, ranging from 5.47 L (100% SoC) to 7.10 L (127% SoC). In 2023 Ubaldi *et al.* reported detailed analysis of TR behaviours for a single NCA cell, this included work in both inert (N_2_) and air atmospheres.^23^ Lammer *et al.* analysed the characteristics of failure events involving three 18 650 NCA cells of similar capacity (3.1–3.5 Ah).^24^ In this work constant gas volume, gas composition and temperature measurements were made. Although the cells were of comparable capacity and the same cathode chemistry there were variations in their behaviour during failure.

Here, we add to the body of work relating specifically to NCA based cells and characterising the outcomes of their failure. We have probed the behaviour of NCA cells of differing capacities, from a range of manufacturers in terms of the volume of gas released, the composition of said gas and the temperature profiles observed during TR.

### The cells tested

The cells used in this work are eight commercially available lithium ion batteries, five of which are from the same manufacturer (A) and three from other individual manufacturers (B, C and D). They offer a range of capacities but all have lithium–nickel–cobalt–aluminium oxide (NCA) cathode chemistries as confirmed by X-ray fluorescence (XRF) (Table 1).

**Table 1:** Cell name (X–Y; X = manufacturer Y = capacity in Ah), format, nominal capacity, typical mass of cells, average measured capacity, average measured internal resistance and values for percentage nickel, cobalt, aluminium and manganese as determined by XRF

Cell name	Cell format	Nominal capacity/Ah	Average measured mass of cell/g	Average measured capacity/Ah	Average internal resistance/mΩ	Cathode composition determined by XRF/%			
						Nickel	Cobalt	Aluminium	Manganese
A-2	18 650	2	46.5	2.02	7.7	87.6	10.4	1.0	0.0
A-3i	21 700	3	67.8	3.00	2.9	87.6	10.3	1.0	0.0
A-3ii^a^	18 650	3	45.8	3.03	9.7	87.5	10.7	0.9	0.0
A-4	21 700	4	67.9	4.00	4.3	88.1	10.4	0.8	0.0
A-5	21 700	4.9	68.3	4.93	9.1	88.3	10.3	0.9	0.0
B-4.2	21 700	4.2	66.0	4.08	4.8	87.5	10.7	0.8	0.0
C-4	21 700	4	67.0	3.95	3.6	81.9	10.4	6.8	0.0
D-5	21 700	5	68.0	4.93	8.6	88.2	9.6	1.1	0.0

a Tested in previous work.

The selection of cells allow comparisons to be made between cells of different capacities and from different manufacturers at 100% SoC, and also comparison of cells from the same manufacturer with varying capacities at a range of SoC and amount of charge (AoC). In this work AoC is used alongside SoC as the dataset includes cells of differing capacities but are tested at comparative amounts of charge. For example a 3 Ah cell charged to 100% SoC, 3 Ah AoC, can be compared with a 5 Ah cell charged to 60% SoC, also 3 Ah of stored energy. Using both SoC and AoC may allow a distinction to be drawn as to whether the amount of stored energy (AoC) is a more or less accurate determinant of cell failure behaviour regardless of overall cell capacity. Or if % SoC is a more suitable metric to frame variations in LIB failure characteristics.

The cells tested in this work are named according to their manufacturer (A, B, C, D), which have been anonymised, and by their capacity in Ah (Table 1). In the dataset are two 3 Ah cells from the same manufacturer (A). A-3ii is data from previously published work carried out in this laboratory and is included here for comparison purposes.^13^A-3ii has the same capacity and chemistry as A-3i but varies in the size of the cell casing.

The cells selected, their nominal capacities as per the manufacturer's datasheet, typical mass of each cell, average measured capacities, average internal resistances and cathode composition as determined by XRF are shown below (Table 1). It is noteworthy that capacity does not affect mass as both 18 650 cells (A-2, A-3ii) are similar, likewise all 21 700 cells (A-3i, A-4, A-5, B-4.2, C-4 and D-5) are comparable in terms of mass.

In total 69 cells have been tested to complete failure, for all of which a volume of gas has been determined. For 52 of the cells bulk gas composition has been determined. At least two tests were carried out for each cell at each AoC. A copy of the full dataset, which includes all relative gas composition, maximum recorded cell surface temperature and gas volumes, where acquired, is detailed in Table 2.

**Table 2:** All tests carried out for each cell type, AoC & SoC, volume of gas released by failure, volume of gas produced as a function of AoC (L Ah^−1^) and of typical cell mass (L g^−1^), maximum cell temperature recorded, relative gas composition of H_2_, CO_2_, CO, C_2_H_6_, C_2_H_4_, C_3_H_8_, C_3_H_6_ and CH_4_

Cell	Failure method	Atmosphere	AoC/Ah	SoC/%	Volume of gas/L	L Ah^−1^	(L g^−1^) × 10	Max. temp./°C	Relative composition/%							
									H_2_	CO_2_	CO	C_2_H_6_	C_2_H_4_	C_3_H_8_	C_3_H_6_	CH_4_
A-2	EH	Ar	1.0	50	2.13	2.13	0.46	570	18.8	34.1	21.3	1.0	2.8	10.7	3.2	8.1
	EH	Ar	1.0	50	2.26	2.26	0.49	526	18.5	29.7	25.4	−0.8	5.6	9.1	4.2	8.2
	EH	N_2_	1.0	50	2.26	2.26	0.49	472	17.3	28.5	32.9	0.6	3.1	7.5	3.0	7.1
	EH	Ar	2.0	100	4.23	2.12	0.91	232	26.2	24.2	29.7	1.5	7.6	1.3	1.2	8.3
	EH	Ar	2.0	100	4.42	2.21	0.95	224	32.8	19.1	32.1	1.1	8.0	1.1	1.1	4.7
	EH	Ar	2.0	100	3.74	1.87	0.81	261	31.7	20.5	30.4	2.5	7.5	1.0	1.3	5.1
	EH	N_2_	2.0	100	3.73	1.86	0.80	197								
	EH	N_2_	2.0	100	3.35	1.67	0.72	218								
A-3i	EH	Ar	1.0	33	2.47	2.47	0.36	355	20.6	27.7	22.8	0.6	3.4	10.6	4.0	10.3
	EH	Ar	1.0	33	2.71	2.71	0.40	451	22.5	32.8	20.2	2.8	4.9	7.0	3.2	6.5
	EH	Ar	2.0	67	4.38	2.19	0.65	138	32.8	25.3	24.1	2.1	4.8	1.7	1.9	7.3
	EH	Ar	2.0	67	4.12	2.06	0.61	272	33.0	19.6	31.7	1.6	5.3	1.5	1.7	5.6
	EH	Ar	3.0	100	6.08	2.03	0.90	92.8	30.4	24.4	29.7	2.5	6.0	0.4	0.4	6.1
	EH	Ar	3.0	100	6.74	2.25	0.99	220	32.9	21.8	30.4	2.3	5.8	0.8	0.9	5.1
	EH	Ar	3.0	100	6.33	2.11	0.93	230	31.9	18.0	35.2	1.5	6.4	0.5	0.9	5.7
	EH	N_2_	3.0	100	8.07	2.69	1.19	41.5								
	EH	N_2_	3.0	100	5.68	1.89	0.84	215								
	EH	N_2_	3.0	100	6.28	2.09	0.93	207								
A-3ii	EH	N_2_	3.0	100	4.70	1.57	1.03		22.4	34.2	22.0	2.4	1.9	1.2		16.2
A-4	EH	Ar	1.0	25	2.91	2.91	0.43	174								
	EH	Ar	1.0	25	3.11	3.11	0.46	344	22.7	23.0	33.5	0.4	6.5	3.5	2.9	7.7
	EH	Ar	1.0	25	4.18	4.18	0.62	517	13.1	42.2	28.7	2.1	2.0	3.9	2.4	5.6
	EH	Ar	2.0	50	4.82	2.41	0.71	289	12.0	39.1	33.3	1.2	2.3	3.0	2.5	6.5
	EH	Ar	2.0	50	4.46	2.23	0.66	331	24.9	26.8	30.2	3.0	5.7	1.8	1.9	5.7
	EH	Ar	3.0	75	5.64	1.88	0.83	405	22.5	24.7	34.7	2.8	6.5	1.6	1.1	6.2
	EH	Ar	3.0	75	5.51	1.84	0.81	229	22.1	22.1	37.7	3.4	5.7	1.2	0.8	7.0
	EH	N_2_	3.0	75	6.77	2.26	1.00	674								
	EH	N_2_	3.0	75	7.52	2.51	1.11	363								
	EH	Ar	4.0	100	7.82	1.96	1.15	170	23.1	22.3	41.6	1.2	5.1	0.7	0.4	5.7
	EH	Ar	4.0	100	9.69	2.42	1.43	587	30.8	19.2	36.0	1.1	7.4	0.5	0.5	4.4
	EH	Ar	4.0	100	7.38	1.84	1.09	353	28.5	21.1	38.8	1.1	4.0	0.4	0.4	5.7
	EH	Ar	4.0	100	7.79	1.95	1.15	315	27.6	20.1	40.6	1.4	3.6	0.5	0.4	5.8
	EH	N_2_	4.0	100	8.71	2.18	1.28	353								
	EH	N_2_	4.0	100	8.15	2.04	1.20	352								
A-4 NP	NP	Ar	4.0	100	8.89	2.22	1.31	465	24.1	24.1	35.5	1.9	7.3	0.4	0.8	5.9
	NP	Ar	4.0	100	9.63	2.41	1.42	665	23.2	23.1	38.3	2.4	6.7	0.4	0.7	5.1
	NP	Ar	4.0	100	9.34	2.34	1.38	526	23.9	25.2	36.9	0.7	7.4	0.4	0.7	4.7
A-5	EH	Ar	1.0	20	2.78	2.78	0.41	530	25.8	30.3	21.1	1.8	3.4	6.0	2.7	8.9
	EH	N_2_	1.0	20	2.24	2.24	0.33	234	21.7	33.2	17.7	3.1	2.9	8.0	2.9	10.7
	EH	Ar	1.0	20	3.06	3.06	0.45	527	30.1	23.9	23.4	0.9	5.0	4.1	2.5	10.1
	EH	Ar	2.0	40	5.39	2.70	0.79	405	19.9	26.2	40.7	0.1	4.1	1.5	1.1	6.5
	EH	Ar	2.0	40	4.93	2.47	0.72	284	14.9	43.2	22.9	2.6	4.0	3.9	2.0	6.6
	EH	N_2_	2.0	40	4.71	2.35	0.69	526	16.9	30.5	34.9	1.6	4.9	3.3	1.7	6.3
	EH	Ar	3.0	60	5.15	1.72	0.75	222	24.1	18.4	44.5	3.1	3.8	1.2	0.6	4.3
	EH	Ar	3.0	60	6.07	2.02	0.89	229	19.0	28.6	35.4	2.3	5.0	2.5	1.3	6.0
	EH	Ar	3.0	60	5.73	1.91	0.84	671	20.5	23.0	37.3	4.3	5.0	2.5	1.5	6.0
	EH	Ar	3.0	60	4.53	1.51	0.66	190	34.2	23.7	26.8	0.4	4.2	2.7	0.7	7.2
	EH	N_2_	3.0	60	6.62	2.21	0.97	716								
	EH	N_2_	3.0	60	6.30	2.10	0.92	408								
	EH	N_2_	3.0	60	6.36	2.12	0.93	649								
	EH	Ar	4.0	80	5.90	1.48	0.86	318	23.3	26.9	38.6	0.7	4.3	1.1	0.4	4.7
	EH	Ar	4.0	80	6.82	1.70	1.00	334	25.2	24.3	36.3	1.5	4.6	1.5	0.5	6.2
	EH	Ar	4.0	80	8.62	2.15	1.26	738	26.5	24.0	38.7	0.1	4.2	0.5	0.3	5.6
	EH	N_2_	4.0	80	7.70	1.93	1.13	616								
	EH	N_2_	4.0	80	7.23	1.81	1.06	355								
	EH	N_2_	5.0	100	7.29	1.46	1.07	259	27.1	30.7	31.2	1.7	2.7	0.7	0.3	5.6
	EH	N_2_	5.0	100	6.54	1.31	0.96	195	19.5	31.9	36.6	2.5	2.9	0.6	0.2	5.8
	EH	Ar	5.0	100	6.96	1.39	1.02	307	24.4	27.4	36.7	2.9	2.3	0.5	0.2	5.6
	EH	N_2_	5.0	100	6.96	1.39	1.02	298								
	EH	N_2_	5.0	100	5.82	1.16	0.85	350								
B-4.2	EH	Ar	4.2	100	7.48	1.78	1.13	518	21.6	16.6	34.9	2.7	4.1	14.2	1.3	4.7
	EH	Ar	4.2	100	5.82	1.39	0.88	389	18.6	17.3	37.7	1.3	3.5	17.0	1.2	3.4
	EH	Ar	4.2	100	8.38	2.00	1.27	611	21.7	16.1	35.6	2.1	3.7	15.2	1.2	4.4
C-4	EH	Ar	4.0	100	9.72	1.94	1.45	513	23.9	19.4	37.6	1.7	9.5	1.0	0.6	6.4
	EH	Ar	4.0	100	8.60	1.72	1.28	352	27.6	19.5	36.1	2.0	7.4	0.6	0.7	6.1
	EH	Ar	4.0	100	9.33	1.87	1.39	589	30.4	19.5	34.1	1.6	7.8	0.5	0.5	5.7
D-5	EH	Ar	5.0	100	11.17	2.23	1.64	461	31.2	19.9	40.6	0.1	2.8	0.4	0.3	4.7
	EH	Ar	5.0	100	8.33	1.67	1.23	245	29.2	24.6	36.9	1.7	2.0	0.3	0.1	5.3
	EH	Ar	5.0	100	8.02	1.60	1.18	222	30.1	25.7	36.4	1.2	1.2	0.4	0.2	4.8

## Method

The pressure vessel (PV) and methodology outlined by Abbott *et al.*^25^ for carrying out cell failure experiments and gas analysis has been used. Similar enclosed testing systems that are designed to withstand and analyse LIB failure are also being deployed in other research groups.^26–28^

NCA cathode chemistry was determined by teardown of cells in a glovebox filled with an argon atmosphere. Electrodes were separated from other cell components and the cathode washed with dimethyl carbonate three times. The cathodes were then analysed by XRF (Niton XL3t GOLDD+) to confirm the presence of nickel, cobalt and aluminium and confirming the absence of manganese (Table 1).

Cells were charged to the requisite SoC using charge/discharge protocols within the recommended parameters set out in the manufacturer's datasheet. All plastic wrappings on the exterior of the cell were removed. A type-N thermocouple was fixed to the cell surface using Kapton tape. Cells were secured in place using the a cell holder housed inside a baffle box (to avoid flame impingement on the inside of the PV), this was placed inside the PV. The PV was sealed and the atmosphere inside the PV was changed by cycling between ambient pressure and approximately ambient pressure +2 bar by the addition then venting of either nitrogen or argon. The first fill was held at +2 bar for six minutes and the pressure monitored to ensure there were no leaks in the PV. The fill–vent cycle was repeated so that a total of seven cycles were completed. In final gas analysis by MS, O_2_ was observed in the range of 0–5% and not accounted for in the final relative gas composition calculations.

After a final venting to ambient pressure, LIBs were heated until TR was observed. For external heat tests, a 2 × 2′′ 10 Ω adhesive heater (OMEGA KHLVA 202-10/P) was applied directly to the surface of the cell can prior to sealing the PV. Heating was achieved by applying a constant voltage of 24 V DC and a current of approx. 1.2–1.3 A (*ca.* 30 W electrical power). The heating profile in Fig. 1 is typical of the heating rate and onset of venting and TR for the cells in this work. In the case of A-4 NP failure was achieved by nail penetration. For nail penetration tests, cells were secured and were pierced by a hardened stainless steel nail (100 mm length, 3 mm diameter with a 40° point) using a 100 mm stroke actuator, powered by 12 V DC power supply giving an unhindered stroke speed of 10 mm s^−1^. The PV rig is contained inside a blast cell and all tests were controlled and monitored remotely.

A fan (24 V, 35 mm diameter) inside the PV was operating throughout all tests and gas sampling stages to ensure a homogenous atmosphere in the PV. TR was determined to have occurred when a sudden increase in temperature and pressure was observed by live monitoring (NI FlexLogger software) of the cell surface temperature, PV internal ambient temperature and the internal pressure of the PV. Both ambient and cell surface temperature and internal PV pressure were recorded throughout the test. Heating was stopped as soon as TR was observed.

After ambient temperature had decreased sufficiently (below 30 °C) a gas sample was taken by allowing gas to flow into a 5 L Tedlar sealable gas bag *via* a gas line fitted to the PV. At least two tests with concordant volumes and relative gas compositions were obtained for each cell at each SoC.

Gas volumes were determined using the ideal gas law equations (eqn (1) and (2)) and corrected for standard temperature and pressure (eqn (3)). The internal volume of the vessel (*V*_vessel_) accounts for the space occupied by test apparatus.

1 *pV* = *nRT*

2
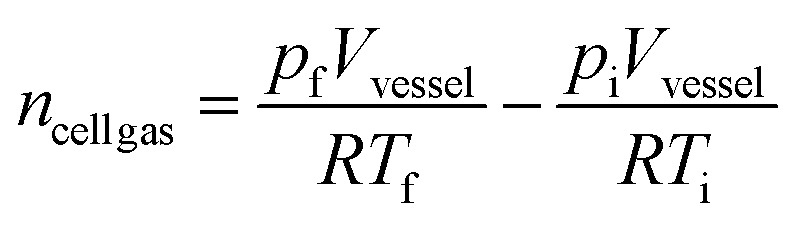
3
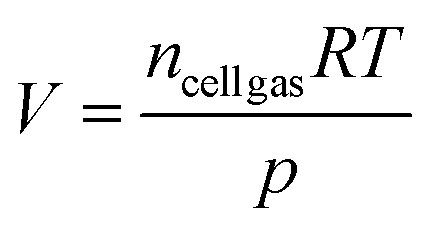


Gas samples were analysed for their composition by MS using a HIDEN (HPR20) as per the method described by Abbott *et al.*^25^ All graphs and discussions regarding volume and relative composition of gases use the average values for each cell at each SoC.

## Results and discussion

### Volume of gas produced during LIB failure

#### Volume of gas produced at 100% SoC

**Fig. 2:**
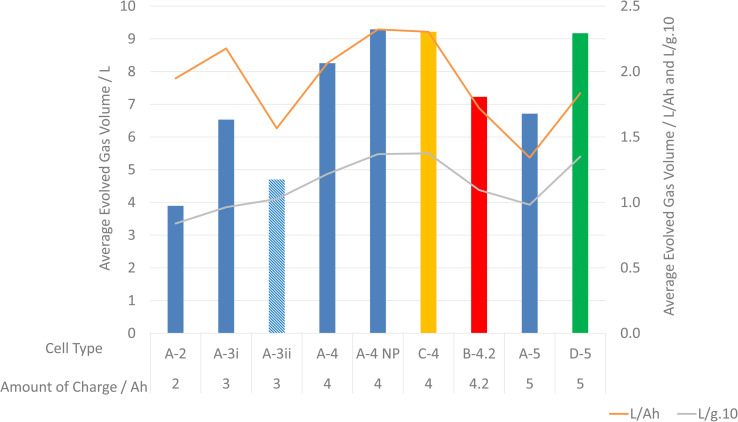
Graph of average volume of gas evolved for each cell at 100% SoC, with volume also expressed in terms of L Ah^−1^ and as a function of the typical mass of each cell, (L g^−1^) × 10.

One of the ways in which the volume of gases emitted during LIB failure has been rationalised is by comparing SoC with gas volumes.^25,29^ In the first instance the volume of gas produced is analysed for each cell at the same SoC. The average volume of gas released at 100% SoC for each of the cells is shown below along with volume expressed as a function of AoC (L Ah^−1^) and as a function of typical mass of each cell (L g^−1^) (Fig. 2). Somewhat predictably, smaller capacity cells A-2, A-3i, A-3ii produce the lowest volume of gas at 100% SoC. However, there is not a universal increase in volume of gas with higher capacity. B-4.2 and A-5 produce lower volumes of gas compared to smaller capacity cells A-4, A-4 NP, C-4. D-5 is the other 5 Ah cell in this set and is closest to A-4 NP and C-4 in terms of volume of gas released but is lower in L Ah^−1^ given the higher capacity of D-5. Seemingly, cell format is the clearest differentiating factor in this dataset with both 18 650 cells (A-2 and A-3ii) producing much less gas than all 21 700 cells (A-3i, A-4, A-5, B-4.2, C-4 and D-5). For the two 18 650 cells, A-3ii produces more gas overall than A-2, but A-2 produces more in terms of L Ah^−1^. Across the 21 700 cells there is not a discernible trend as AoC and capacity do not necessarily lead to a greater or lesser volume of gas. The 21 700 cells produce varying volumes of gas and vary in terms of L Ah^−1^. For all the cells the volume of gas at 100% SoC volume produced during TR is nuanced and seems to be largely cell dependant.

The method of failure has an interesting effect on the volume of gas produced. A-4 when failed by nail penetration (A-4 NP) produced a greater volume of gas compared to all the cells in the dataset. Nail penetration has been shown to be a nuanced method of failure and induces instantaneous TR in some cells.^25^ Nail penetration and external heat methods have been compared previously. Diaz *et al.* observed that 2.5 Ah pouch cells produced less gas when failed by nail penetration compared to heating at 100% SoC. Interestingly, no TR was observed at 50% SoC for nail penetration but was observed with heating as the method of failure.^30^ Essl *et al.* made observations similar to this work, when two different NMC pouch cells were failed by heating or nail penetration at 100% SoC more gas was released by nail penetration (Cell 1: 1.56 L Ah^−1^ EH, 1.71 L Ah^−1^ NP and Cell 2: 1.56 L Ah^−1^ EH and 1.77 L Ah^−1^ NP).^31^

It is unclear the extent that cathode composition has an effect on volume of gas produced as this is only one of many design considerations involved in cell manufacture. However, it is noteworthy that C-4 has more aluminium content in its cathode than the other cells in this series (Table 1) and produced the most gas when failed by heating as well as the most gas in terms of L Ah^−1^. Cathode chemistry is one of many highly tuned aspects of cell design that form the finished product.

This work shows that cells of comparable capacities can vary significantly. For example, A-5 at 100% SoC produces less gas than each of the 4–5 Ah cells and is closest to A-3i in terms of volume of gas released (Fig. 2). For cells in this work, understanding the volume of gas produced during failure, studying each individual cell type is more useful form a safety perspective compared to making broad assumptions based other parameters.

#### Volume of gas produced at differing AoC

**Fig. 3:**
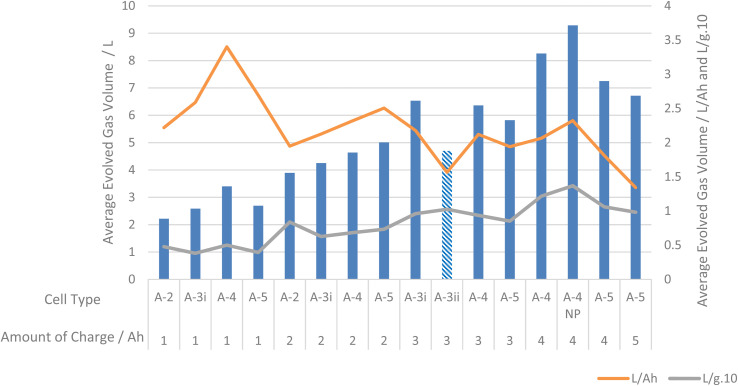
Graph of average evolved volume of gas for each cell from manufacturer A and L Ah^−1^ and (L g^−1^) × 10.

Cells from manufacturer A were tested at varying AoC. As cells from manufacturer A increased in AoC the volume of gas evolved from LIB failure also increased (Fig. 3). When volume is expressed in terms of L Ah^−1^ there is, in general, a decrease in the volume of evolved gas as a function of AoC As previously identified, the largest average volume of gas released was from cell A-4 when failed by nail penetration (A4-NP) charged to 4 Ah. At 1, 3 and 4 Ah cell A-4 also produced the largest average volume of gas compared to other cells with the same AoC. Interestingly, at 2 Ah cell A-5 produced the largest average volume of gas. This is the only AoC that A-5 produced the most gas, at all other AoC cell A-5 produced the lowest average volume of gas (Fig. 3).

Estimating amounts of gas released as a function of SoC and AoC of a cell can be used to give only an impression of the amount of gas that will be released from LIB failure. The manner of abuse that has led to LIB failure can also be somewhat taken into account when estimating volumes of gas released. Generally, the larger the LIB and the more stored energy in an LIB the greater the volume of gas. However, it should be noted that such assertion has considerable limitations.

Whilst for critical applications, measurements of volumes should be preferred, this work gives an indication of expected volumes of gases evolved during LIB failure. At 100% SoC, representing a worst case scenario under normal operating conditions, one can expect approximately 2 L Ah^−1^ of gas to be evolved. It should also be noted that lower SoCs will not reduce the amount of gas evolved to zero. As shown in Fig. 3 at lower SoCs the amount of gas evolved in terms of L Ah^−1^ can be >3 L Ah^−1^.

The volume of gas released will also be effected by the amount of O_2_ present, as many combustion reactions involving LIB failure gases will consume O_2_.

### Relative composition of LIB failure gases

#### Gas compositions of cells from different manufacturers at 100% SoC

The mixture of gases evolved during the failure of LIBs are the products of a significant number of complex degradation processes.^32,33^ The impact of gas composition at varying SoC has been studied.^32^ As we have tested cells of varying capacities 100% SoC was chosen as an appropriate SoC to compare relative compositions of the gaseous products of LIB failure. We have focussed on the gases CO_2_, CO, H_2_, CH_4_, C_2_H_6_, C_2_H_4_, C_3_H_8_ and C_3_H_6_. In this work tests to characterise the gaseous products of LIB failure were carried out in either nitrogen or argon. Had oxygen been present gas composition would likely be different.^34^ The aim of this work is not to determine the origins of the gases but to make better first assessments of the flammability of the failure gases produced by TR.

**Fig. 4:**
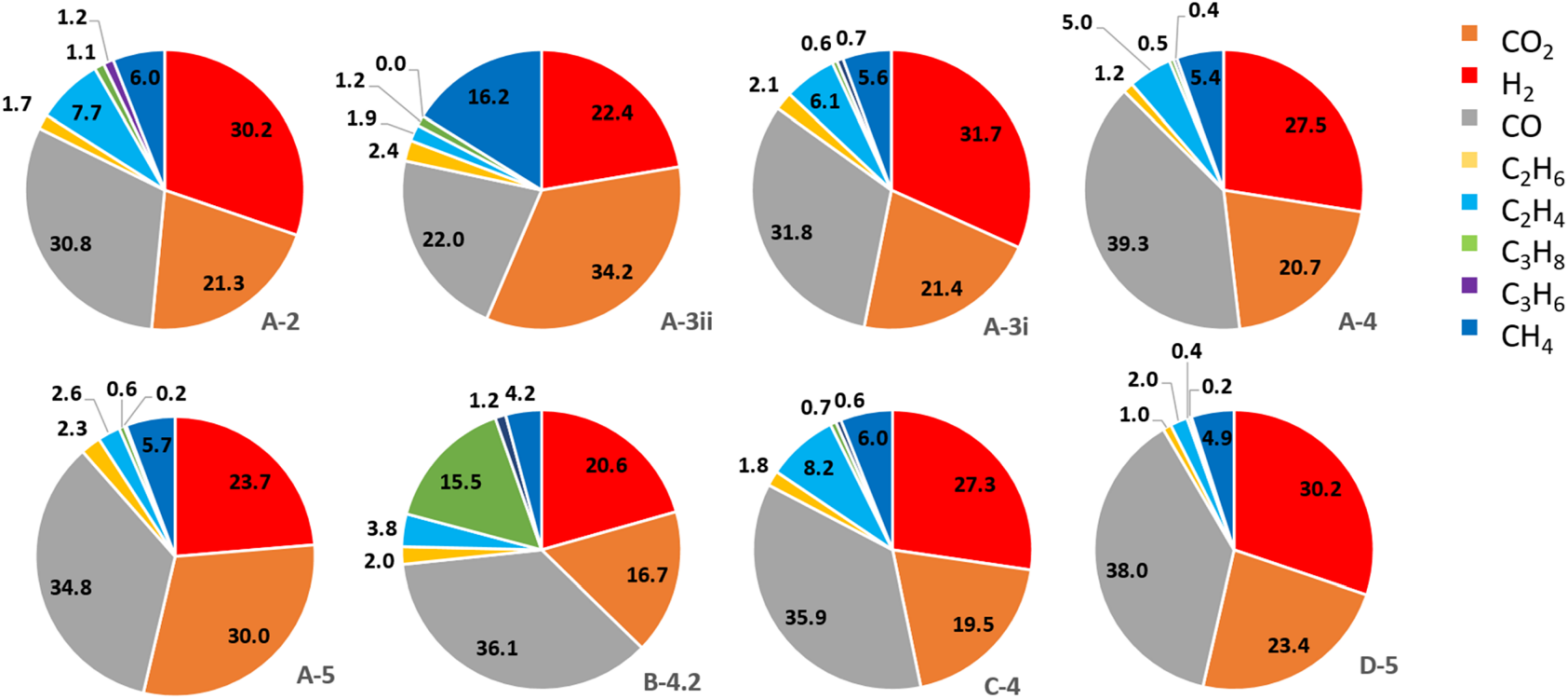
Pie chart showing the relative amount of CO_2_, H_2_, CO, C_2_H_6_, C_2_H_4_, C_3_H_8_, C_3_H_6_ and CH_4_ for each of the cells at 100% SoC.

As with the results for volume of gas, the relative gas composition of A-3ii at 100% SoC has also been included from previous work. In that report propene was not included in the relative gas composition.^13^ The relative gas composition data for cells failed at 100% SoC is shown below (Fig. 4).

In all cases the main products, of the eight gases tested for, produced by LIB failure are H_2_ (21–31%), CO (22–39%) and CO_2_ (20–34%) with smaller amounts of mixed hydrocarbons (9–27% total). There are two cells that produce a greater amount of one hydrocarbon. Firstly, B-4.2, which produced 15.5% propane. In all of the other cells tested propane is a minor product of LIB failure. B-4.2 also produced the least CO_2_ (16.7%) at 100% SoC and has the highest % total hydrocarbon content. Secondly, A-3ii produced 16.2% CH_4_ and also produced the most CO_2_ (34.2%). The understanding of the composition of LIB failure gases can be used as a crucial tool in assessing the risk these emissions pose in terms of flammability and toxicity.

The relative composition of flammable components from this current campaign of testing (excluding A-3ii) varies from approximately 70% (A-5) to 83% (D-5). D-5 also shows much less relative hydrocarbon concentration compared to the other cells in this series but is amongst the more significant producers of hydrogen (30%). It is unsurprising that there are such variations in the hydrocarbon composition of the failure gases given the array of electrolyte solvent mixes that are used in LIB manufacture. The variations in cathode composition do not appear to be manifested in the composition of failure gases. C-4 having a higher aluminium content produces comparable levels of the three majority component gases. There is slight nuance in the amount of C_2_H_4_ produced however the overall hydrocarbon component is comparable to other cells in the series.

#### Gas composition analysis of cells from the same manufacturer at varying SoC/AoC

In general, for cells from manufacturer A the relative amount of CO and H_2_ increases as AoC increases, whilst there is a decrease in CO_2_ and hydrocarbon content with increasing AoC. At higher SoC/AoC for each cell CH_4_ and C_2_H_4_ tend to be the predominant hydrocarbon components. C_3_H_6_ and C_3_H_8_ are present in greater proportion at lower SoC/AoC but diminish as SoC/AoC increases. It is plausible that longer chained hydrocarbon fragments are further decomposed in TR events involving cells with higher SoC. However, the exact mechanism of formation for these products is an area of study in of itself and is beyond the scope of this work.^33,35–38^

**Fig. 5:**
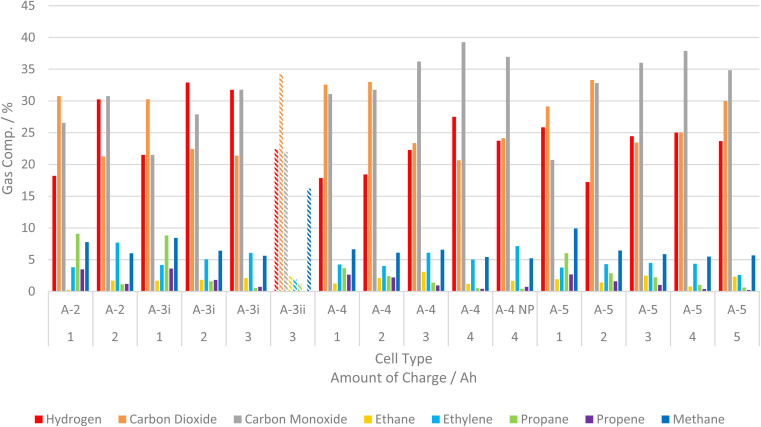
Graph of relative gas composition for all cells from manufacturer A at each SoC tested, bars for A-3ii are hatched as they are results from previous work.^25^

A-4 has been made to fail by both heating and nail penetration (NP). For A-4 NP there was less hydrogen but greater relative amounts of CO_2_ and hydrocarbons (Fig. 5), as well as the increased volume of gas as discussed previously.

A-3i produces a much greater relative amount of H_2_ than other cells in the series at comparable AoC, except at 1 Ah. At this AoC A-5 produces a significant amount of H_2_ and a similar average volume to A-3i (Fig. 5). For cells from manufacturer A at <50% SoC there is relatively more CO_2_ than CO. Once SoC is >50% there is a change in the relative amounts of CO and CO_2_ and CO becomes the major component. The production of CO as a major component has implications for both the flammability and toxicity of LIB failure gases. Xu *et al.* observed a similar change in NMC failure gases; increasing CO and decreasing CO_2_ production as SoC increased.^28^

Considering each cell type individually: for A-2 the proportion of flammable gases (all excluding CO_2_) increases as AoC changes from 1 to 2 Ah. For A-3i the flammability increases as AoC changes from 1 to 2 Ah but there is little difference between 2 and 3 Ah. For A-4 the proportion of flammable components is similar at 1 and 2 Ah then increases as AoC changes from 2 to 3 Ah. However, the proportion of flammable components is similar between 3 and 4 Ah. For A-5 the proportion of flammable components increases moving from 2 to 3 Ah but 1–2 Ah and 3–5 Ah are somewhat comparable.

In general, the observed changes in the proportion of flammable components are subtle. However, any change in flammability profiles of LIB vent gases has clear implications when considering the consequences of LIB failure events. Predicting the profile of LIB failure gases is extremely difficult even under tightly managed test conditions. Extrapolating to real world scenarios is compounded given the added number of variables. However, an appreciation of the hazards that LIB failure may present is a crucial consideration when designing equipment and facilities that utilise LIBs.

#### Changes in observed maximum cell surface temperature with SoC

**Fig. 6:**
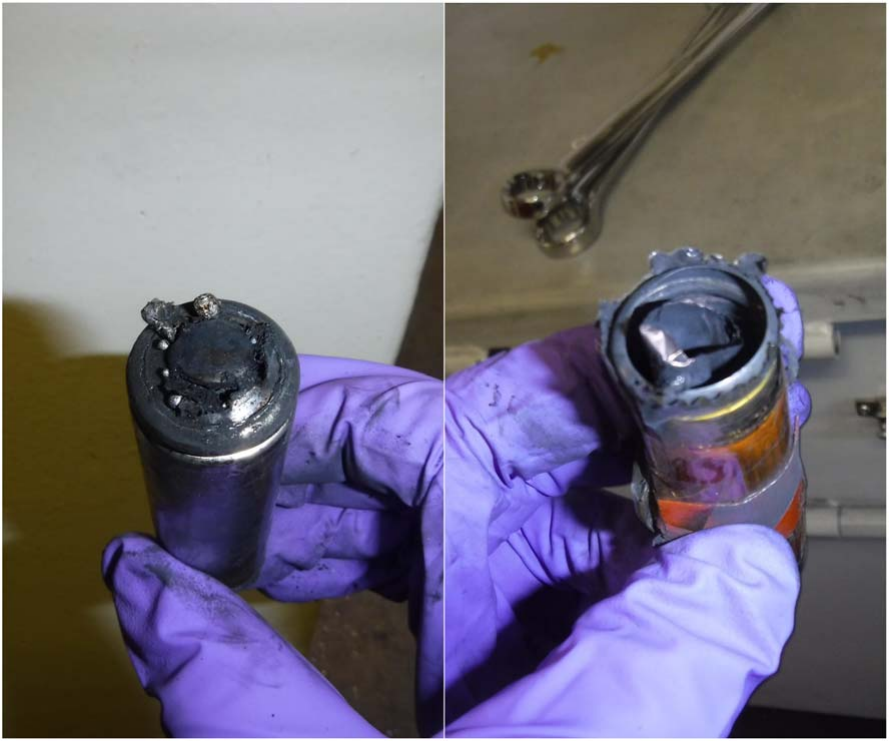
Comparison of two A-3i cells after failure by external heating at 67% SoC (left) and 100% SoC (right).

Cell surface temperature was monitored throughout each test to ensure progression of heating and to monitor for the onset of TR, the results are included in Table 2. At higher SoC TR events tend to become more extreme. Paradoxically, during this work maximum cell surface temperatures were frequently observed to be lower at higher SoCs. For example, A-2 at 50% SoC recorded maximum cell surface temperature values of 472–570 °C but at 100% SoC maximum cell surface temperatures were in the range of 197–261 °C. On first inspection this may seem counterintuitive but the increased ferocity of TR at higher SoCs is likely responsible for such trends within the dataset. More extreme events more frequently result in the contents of LIBs being ejected and therefore the decomposition processes cannot be detected by thermocouples on LIB surfaces. An increase in ferocity of TR can also result in thermocouples becoming disconnected from LIB surfaces, again giving rise to lower maximum cell surface temperature measurements (Fig. 6).

**Fig. 7:**
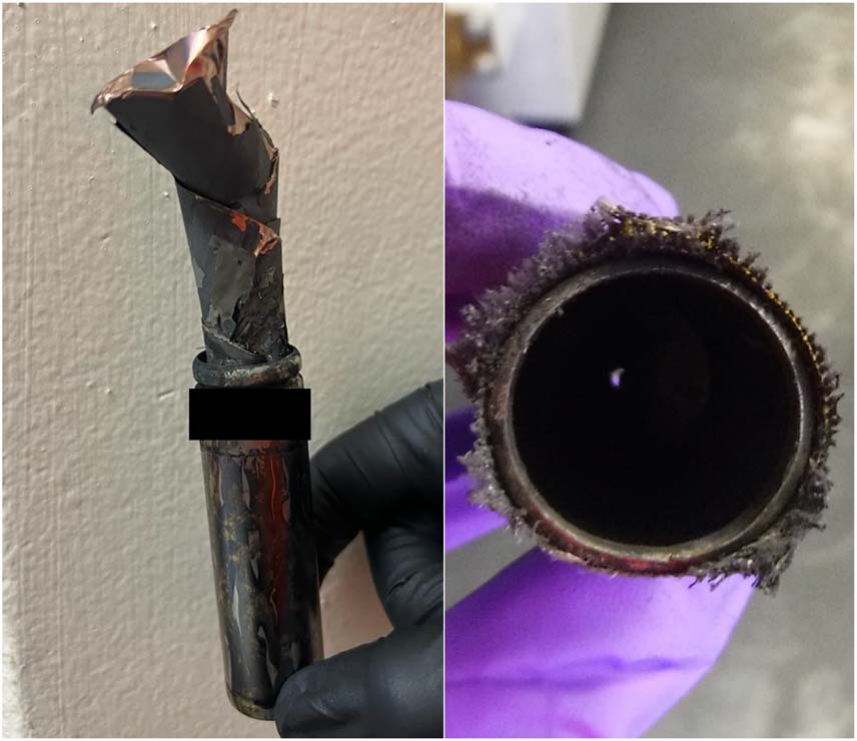
Comparison of two A-4 cells after failure by external heating at 50% SoC (left, side aspect) and 100% SoC (right, vertical aspect).

**Fig. 8:**
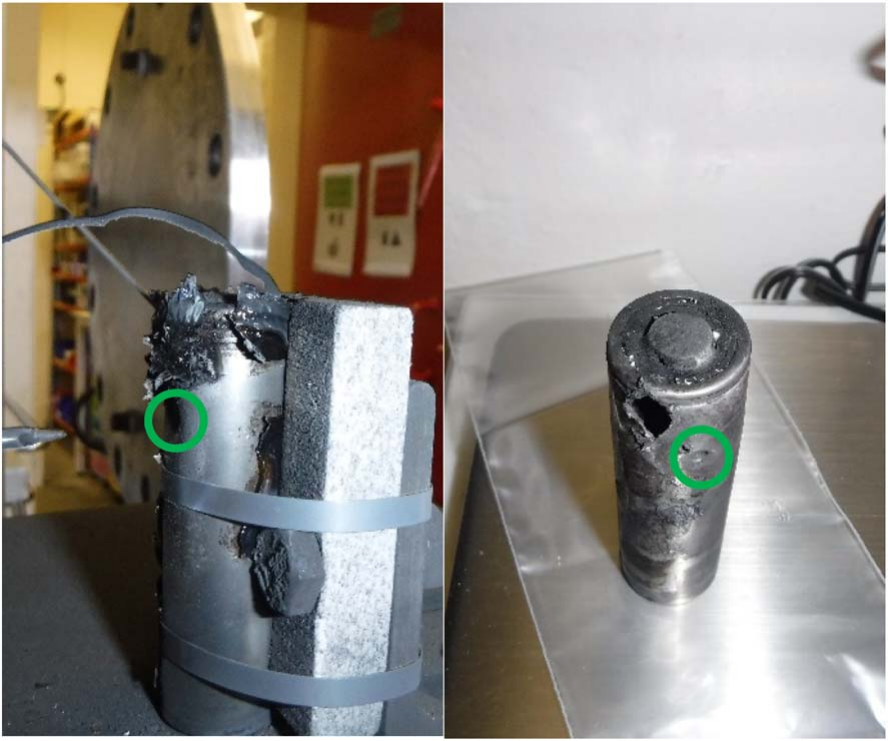
Images of the effects of cell failure after a A-4 NP test, the green circle indicates the point of entry of the nail.

The method of failure also has an impact on the maximum temperature recorded. The maximum temperature readings were higher in A-4 NP tests (465–665 °C, average = 552 °C from three tests) than that of A-4 (170–587 °C, average = 355 °C from six tests). This may be due to the absence of a slow internal pressure build up during nail penetration compared to external heating. Interestingly for A-4 NP the cell casing did not open at the positive terminal/vent cap but failed at several points along the length of the casing (Fig. 8). The observed openings are smaller than the vent cap and would not permit the rolled interior of a typical cylindrical cell to be ejected in a manner shown in Fig. 7.

Here, the analysis of cell surface temperature is only cursory. Other publications have explored such phenomena in greater detail. Cathode composition and its effects on temperature releases during LIB failure were reported by Liu *et al.*^39^ Chen *et al.* carried out investigations into the effects of SoC on temperature and rupture of cells during NP abuse tests.^40^ Perea *et al.* reported the effect of SoC on thermal reactions as a result of LIB abuse.^41^ Buckwell *et al.* examined the relationship between maximum cell surface temperature and mass loss during TR, showing lower maximum cell surface temperature values when greater mass losses were seen.^42^ Finegan *et al.* have also used high speed X-ray imaging to record the venting and TR processes of 18 650 cells, which included NCA cells.^43^ In terms of predicting when TR may be about to occur cell temperature measurements can be vital but ought to be considered as one part of a holistic approach to LIB monitoring and LIB failure characterisation.

## Conclusion

### Volume of gas evolved

Existing consensus is supported in that cells at higher SoC produce greater volumes of gas during TR. The initial relationship between volume and SoC/AoC has been probed for both absolute volume and in terms of L Ah^−1^. It was found that, for NCA cells across four manufacturers and eight cell types, approximately 2 L Ah^−1^ of gas is evolved at 100% SoC, but this varies depending on cell type. Volume of gas evolved decreases as SoC/AoC decrease, however some cells may produce significantly more gas in terms of L Ah^−1^ at low SoC. It has also been shown that two cells with the same AoC, regardless of either cells nominal capacity, may produce differing volumes of gas during TR. It has been shown that the amount of gas produced is more dependent on the type of cell rather than solely SoC or AoC. The method of failure has also been shown to have an impact on the volume of gas released, with nail penetration tending to produce more gas for the two permutations (A-4 at 100% SoC) compared here.

### Relative composition of gases evolved

Generally, the relative amounts of H_2_ and CO increase with AoC whilst hydrocarbons and CO_2_ decrease with increasing AoC. Across this dataset, cells sourced from different manufacturers, with different capacities vary in the composition of evolved gases evolved during LIB failure. Typical compositions for gases produced by NCA LIB failure at 100% SoC are around 25–30% H_2_, 30–35% CO, ∼25% CO_2_ and 10–15% mixed hydrocarbons. Although a fuller understanding of LIB failure characteristics can only be achieved by testing the LIB under consideration.

### Cell surface temperature

Cell surface temperature has been used primarily as a means to monitor all tests in this work, but also demonstrated that the method of abuse leads to different modes of failure. Nail penetration leads to near immediate TR. This means that cell casings do not necessarily slowly build in internal pressure, such as in external heating tests, and rupture in areas that are not necessarily the safety vent. This means the gas emission behaviour of cells damaged in this way may be more unpredictable. This has implications when considering the design of systems involving LIBs and tackling the consequences of LIB failure.

From cell surface temperature measurements and post-test observations it has been proposed that cells at higher SoC, when failed by heating, have a greater propensity to eject their contents, consequently leading to lower maximum cell surface temperature readings during TR. This similarly has implications when considering the design of systems that utilise LIBs.

## Data availability

All relevant discussed data has been reported in the main body of this paper and any methods for calculations cited accordingly.

## Author contributions

Philip A. P. Reeve: conceptualization, formal analysis, investigation, visualisation writing – original draft. Jonathan E. H. Buston: conceptualization, funding acquisition, supervision, writing – review & editing. Jason Gill: conceptualization, funding acquisition, supervision, writing – review & editing. Steven L. Goddard: data curation, formal analysis investigation, methodology, validation. Gemma Howard: formal analysis, investigation. Jack W. Mellor: investigation.

## Conflicts of interest

The authors declare that they have no known competing financial interests or personal relationships that could have appeared to influence the work reported in this paper.
